# Multimodal Imaging Approach to Atrial Myxoma to Optimize Surgical Management

**DOI:** 10.7759/cureus.66719

**Published:** 2024-08-12

**Authors:** Vineel Lal, James Harvey, Stephen Kyranis

**Affiliations:** 1 Internal Medicine, Princess Alexandra Hospital, Brisbane, AUS; 2 Radiology, Princess Alexandra Hospital, Brisbane, AUS; 3 Cardiology, Royal Brisbane and Women's Hospital, Brisbane, AUS

**Keywords:** echocardiography-heart failure-valvular heart disease, cardiac magnetic resonance (cmr), 3d-echo, 2d echocardiography, large atrial myxoma

## Abstract

Cardiac masses are rare and of the neoplastic group, myxomas are the most common. An elderly male with a background of multiple myeloma and previous autologous stem cell transplant presented with dyspnea and chest heaviness with a subsequent cardiac mass found. Multimodal imaging, including three-dimensional (3D) echocardiography and cardiac magnetic resonance (CMR) imaging, was utilized to guide the diagnostic process, particularly given the differential of a cardiac plasmacytoma in the setting of multiple myeloma. CMR was used to identify characteristic features of the mass and 3D echocardiography highlighted the anatomical relationships of the mass in relation to surrounding structures to complement surgical planning. The different imaging approaches and clinical decision-making were implemented to aid in definitive surgical management.

## Introduction

Primary cardiac tumors are rare with autopsy findings indicating a prevalence of 0.03% [1]. Of the primary non-embryonic cardiac tumors, myxoma is the most common [2]. The presenting features of cardiac masses vary widely and characteristics such as location, and size can affect symptom manifestations such as exertional dyspnea, chest pain, and thromboembolic phenomenon [3]. Whilst echocardiography remains the initial investigation of choice, the use of cardiac magnetic resonance (CMR) imaging can convey valuable information and aid in excluding differential diagnoses such as thrombi [4]. Transthoracic echocardiography (TTE) is readily available and useful but can be non-diagnostic due to inadequate detail of the cardiac mass, variable success with image procurement due to patient factors, and lack of detail of limited windows [3]. Transesophageal echocardiography (TOE) can provide added information, especially for identifying the relation of atrial masses with surrounding structures [5]. It has benefits over CMR in visualizing mobile masses that are valve adjacent [5]. CMR is not invasive and can offer visualization of the mass, and its location in relation to surrounding structures and help to differentiate a possible cause [3]. The CMR protocol needs to encompass all available sequences [3]. A thrombus, for example, will have a hypotensive border and bright center on late gadolinium enhancement whereas a myxoma will be heterogenous [3]. Histopathology is the definitive diagnostic tool for cardiac masses but utilizing all imaging formats available can augment clinical decision-making when dealing with a cardiac lesion [3]. It is essential to identify concurrent pathologies such as coronary artery disease and valvular pathology with a multimodal imaging approach being invaluable.

## Case presentation

An 80-year-old male presented to the Emergency Department complaining of palpitations and chest heaviness. Their past medical history included type 2 diabetes mellitus on oral agents only, hypertension, and hypercholesterolemia. He also suffered from multiple myeloma, with an autologous stem cell transplant 10 years prior to this presentation. They had remained stable since then, without signs of active disease, and he was on monthly zoledronic acid infusions. Initial observations indicated a blood pressure of 134/70 mmHg, a respiratory rate of 19 breaths per minute, and an oxygenation saturation of 95% on room air. Their heart rate in Emergency was 83 beats per minute (bpm). An ECG showing ST elevation in aVR and widespread ST depression with a right bundle branch block, indicating significant coronary artery disease of the left main coronary artery or proximal left anterior descending artery (LAD) or widespread disease. A clinical exam revealed an ejection systolic murmur loudest in his aortic region with radiation to his carotid arteries, consistent with clinical aortic stenosis.

Initial blood tests revealed a hemoglobin of 142 g/L (120-180 g/L), HbA1c of 7.1% (<6.0%), and a high sensitivity troponin (TnI) of 177 ng/L (<20 ng/L) which rose to 8945 ng/L (<20 ng/L). The other biochemical factors such as kidney function and liver function were within normal limits. They did exhibit an IgG kappa band in his serum and urinary Bence Jones protein was detected, with levels stable with prior assessment. He was treated for non-ST segment myocardial infarction (NSTEMI) with dual antiplatelet therapy and low molecular weight heparin.

He underwent a TTE, CMR, and TOE which revealed a large left atrial mass, and their aortic valve was also shown to be stenosed with moderate to severe disease as indicated by an orifice area of 1.0 cm^2^ (>2 cm^2^) and a mean gradient of 28 mmHg (<25 mmHg). Coronary angiography exposed disease in the LAD, two obtuse marginal branches, and the right coronary artery.

TTE revealed a 3.3 x 3.8 cm mobile mass in the left atrium which appeared to be attached to the interatrial septum (IAS). Their aortic valve was also shown to be stenosed with severe disease as indicated by an orifice area of 1.0 cm2 and a peak gradient of 49 mmHg.

**Figure 1:**
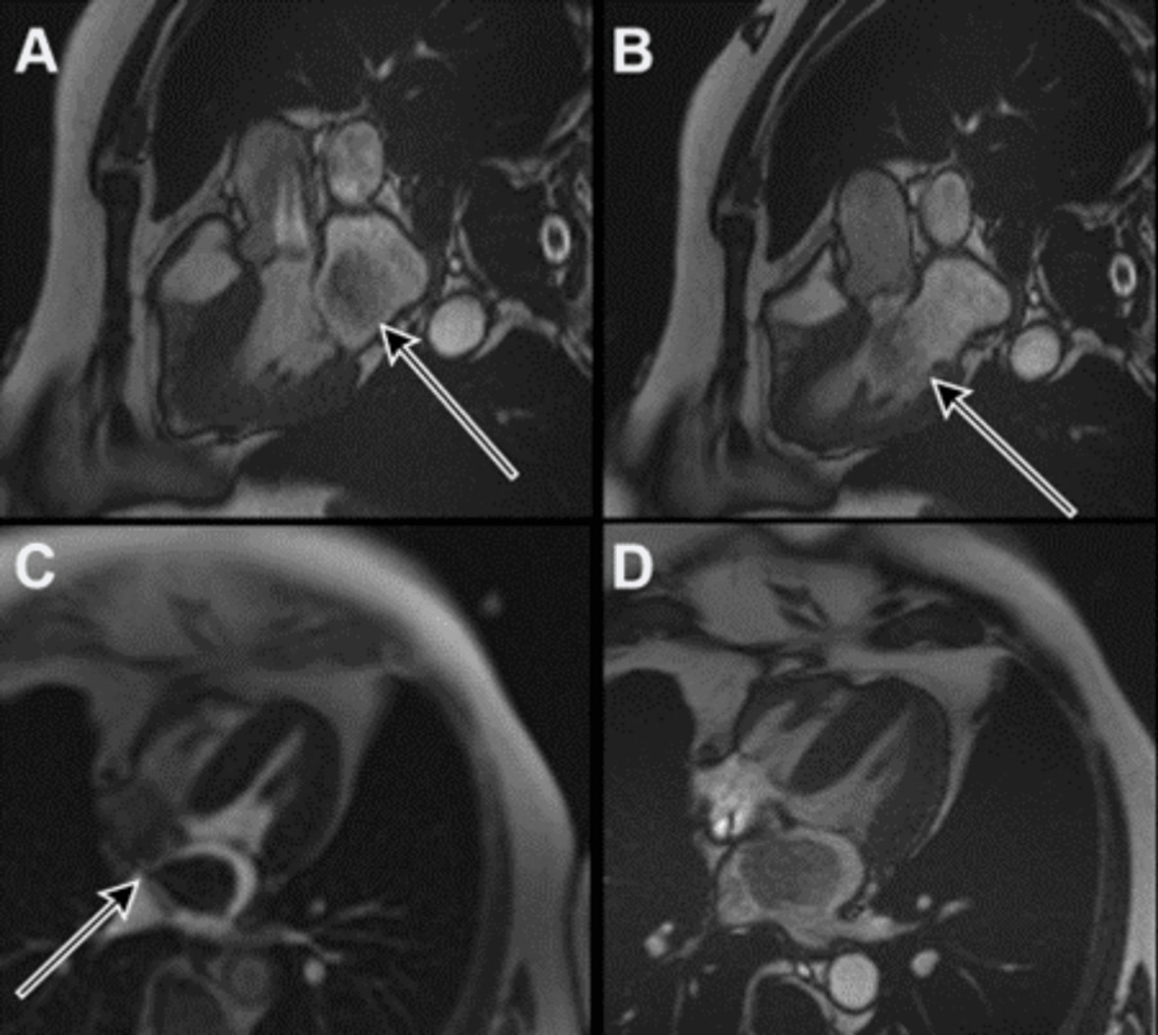
STIR Three-chamber view in systole demonstrating a large mass occupying the left atrium (A; black arrow). During diastole, with T2-STIR, the mobile mass is seen to prolapse through the mitral valve into the left ventricle (B; black arrow). Four-chamber first pass perfusion imaging shows the inhomogeneous filling defect within the left atrium surrounded by contrast (C; black arrow). The mass is seen to attach to the interatrial septum via a thin stalk (arrow). The left atrium is mildly dilated. Four-chamber view 20 minutes post IV gadolinium-chelate injection showing heterogeneous late-gadolinium enhancement (LGE) of the mass (D). STIR: short-tau inversion recovery

CMR was performed with all available sequences. Dynamic cine imaging was used for the cardiac chamber, wall-motion analysis, and valvular analysis. A T2 short-tau inversion recovery (STIR) imaging phase was utilized along with delayed gadolinium-enhancement analysis. This exhibited a 4.1 x 2.8 x 3.2 cm mobile, pedunculated mass in the left atrial which was attached to the IAS by a stalk and prolapsed through the mitral valve with diastole (Figures 1A-1C). There was heterogenous enhancement of this mass with late gadolinium analysis (Figure 1D). Given his history of multiple myeloma, a cardiac plasmacytoma was considered as a differential for this mass. His multiple myeloma was well controlled, and he had no features of active disease, which would include anemia, hypercalcemia, and kidney impairment, it was determined, given the imaging findings, further evaluated in the discussion, and consultation with his Hematologist, that this was unlikely.

**Figure 2:**
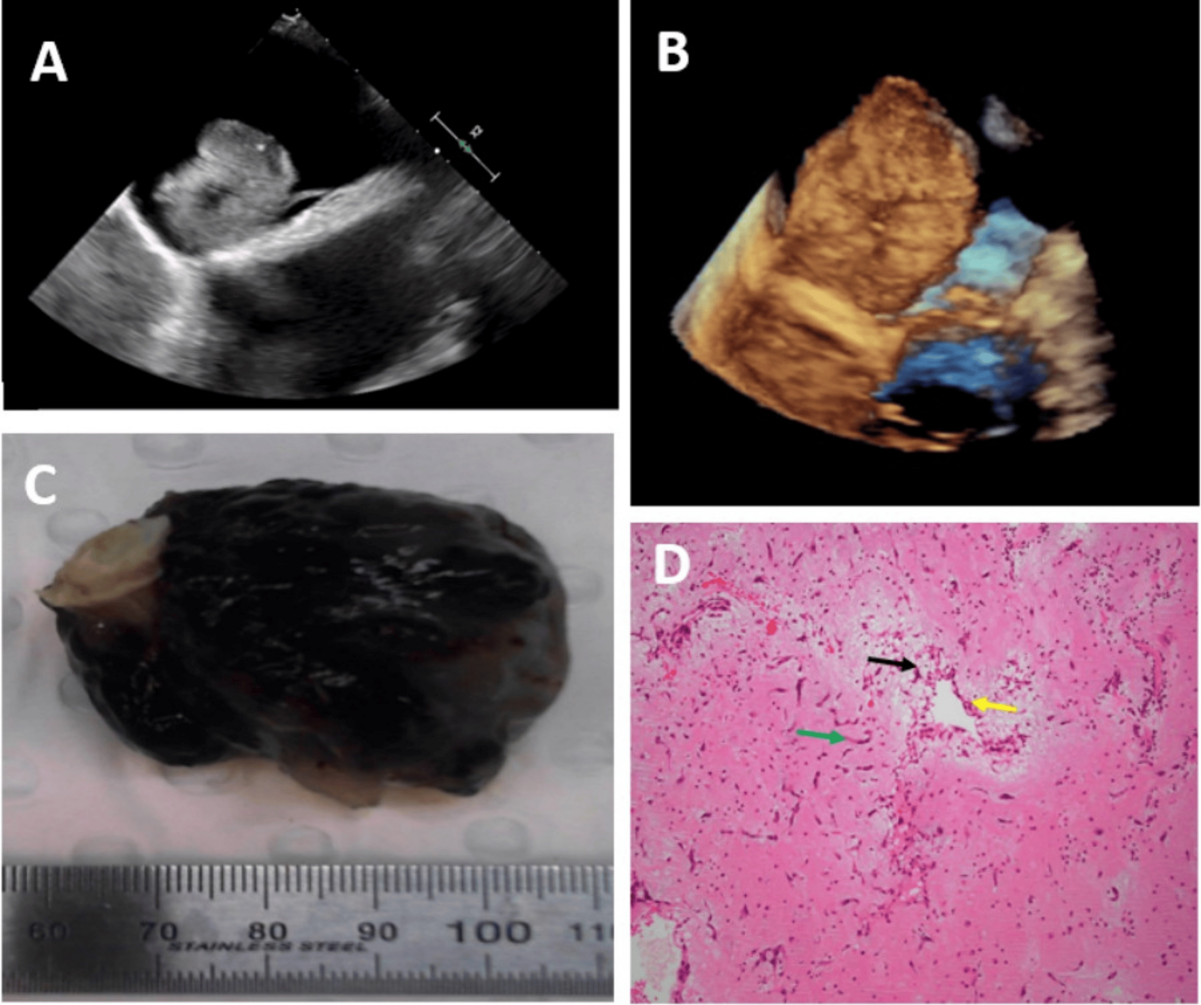
TOE showing a large pedunculated mass in the left atrium (A). 3D reconstruction from TTE views of mass attached to IAS and relationship to pulmonary vein (B). 5.2cm mass excised from left atrium (C). Microscopic analysis showed myxoid stroma (D; black arrow), spindle cells showing pseudovascular channels (yellow arrow), and stellate myxoma cells (green arrow). TOE - Transoesophageal echocardiography, TTE - transthoracic echocardiogram

TOE highlighted a large pedunculated mobile mass with attachment to the interatrial septum (Figure 2A). Three-dimensional (3D) multiplanar reconstruction was utilized to provide greater spatial analysis (Figure 2B). The patient subsequently underwent definitive surgical intervention with an excision of the left atrial mass, a quadruple coronary artery bypass graft (CABG), and a bioprosthetic aortic valve replacement (AVR) with a bioprosthetic valve. Histopathological analysis confirmed the left atrial mass as a myxoma (Figures 2C, 2D). His postoperative course was uneventful, and he remained well in further follow-up.

## Discussion

Myxomas are rare but the most common neoplastic cardiac tumor; metastatic tumors to the heart are approximately 30 times more common [1]. They are more common in women and will present as a mobile heterogenous mass with a narrow stalk [6]. The presenting complaints commonly consist of dyspnea, congestive heart failure, syncope, palpitation, or constitutional symptoms [2]. Around one-third of patients may not exhibit any symptoms at all [2]. 75% of all myxomas are found within the left atrium and 90% occur singularly [7,8]. Within the left atrium, the majority are found at the fossa ovalis [8]. Half of the myxomas will embody a smooth surface and half will be irregular [9]. The complications of myxoma progression include embolic phenomenon, valvular issues such as stenosis and regurgitation, and the development of arrhythmias including atrial fibrillation [2,10].

Differentials such as rheumatic heart disease, infective endocarditis, and connective tissue diseases must be excluded [11]. TTE has a 95% sensitivity in diagnosing myxoma and can provide insight into the location and size of the mass [12]. TOE has a 100% sensitivity and can identify attachment sites and characteristics such as calcifications and vascular features of myxomatous masses [12]. Newer techniques with echocardiography such as 3D reconstruction can provide important information on the spatial aspects of a cardiac mass and anatomical relationships [13]. On echocardiography myxomas and thrombus can appear similar [4]. Other masses that are important to exclude are lipomas, haemangiomas, paragangliomas, sarcomas, etc. [2]. 3D echocardiography is useful in providing detailed analysis of cardiac masses and association with anatomical structures, which is invaluable in the pre-operative planning process [13].

CMR is a significant tool in the diagnosis and workup of a cardiac mass. A myxoma on CMR will appear as a well-defined smooth or lobulated mass that is pedunculated [3]. Signal characteristics do differ [4]. An intermediate signal is often seen on T1-weighted sequences, and a high signal intensity is expressed on T2 sequences [4]. The mixed signal is due to myxoma being a heterogeneous mixture of myxoid and fibrous tissue and hemorrhagic breakdown [4]. With gadolinium administration will show mixed delayed enhancement, which is due to areas of myxomatous and necrotic tissue both being present [4]. With thrombi, late gadolinium enhancement is not seen [4].

Hematological malignancies metastasizing to the heart are rare and only 6.3% of those are plasmacytoma [13]. It was important to exclude plasmacytoma in our patient, given his history of multiple myeloma. One case study of a biopsy-proven plasmacytoma indicated an isointense T1 and T2 signal on CMR [14]. Myxomas on the other hand, like in our case, will be isointense in T1-weighted imaging and hyperintense in T2 imaging [3]. Plasmacytomas will show on imaging as an infiltrative mass, abutting adjoining structures, without a stalk [14]. The imaging features combined with the stable biochemical markers of his multiple myeloma, along with a discussion between cardiology and hematology, indicated plasmacytoma as less likely and therefore treatment such as chemotherapy or radiotherapy was not considered, and surgical excision was favored.

Surgical management is the mainstay of treatment. Outcomes are often favorable with recurrence rates of 1%-3% for non-familial forms of myxoma, 12% for familial forms, and 22% for Carney complex forms [15]. Concurrent pathologies must be excluded to guide adequate and appropriate surgical care. One case series of 11 patients with primary cardiac myxomas found nearly half of them had co-existing coronary artery disease necessitating intervention [8].

On excision and histopathology analysis, a typical appearance of stromal myxoid changes with stellate and spindle cells was seen, indicative of myxoma [16]. A plasmacytoma, on histopathology, would exhibit mononuclear cells embodied by eccentric nuclei [14]. Immunohistochemistry would also reveal CD138 positive staining, which is characteristic of plasma cells and therefore a plasmacytoma.

## Conclusions

Our case highlights the benefit of a multimodal approach to cardiac masses and utilizing newer and different imaging techniques such as 3D echocardiography and CMR. Through the use of multiple imaging formats, along with biochemical analysis and clinical decision-making, we were able to provisionally diagnose our patient with a pathology, myxoma, which was amenable to definitive surgical intervention. This was relevant in this case as the alternative diagnosis of plasmacytoma would require other treatments such as chemotherapy or radiation. It emphasizes that employing all imaging tools can aid in the clinical decision-making process and ensure appropriate care is delivered.
